# Post-COVID-19 Infection With Meticillin-Sensitive Staphylococcus aureus (MSSA) Bacteremia, Discitis/Osteomyelitis, and Diffuse Abscesses: A Case Report

**DOI:** 10.7759/cureus.25824

**Published:** 2022-06-10

**Authors:** Michael Qiu, Deepthi Jayasekara, Arindra Jayasekara

**Affiliations:** 1 Internal Medicine, University at Buffalo, Buffalo, USA; 2 Internal Medicine, Western University of Health Sciences, Pomona, USA; 3 Infectious Disease, Emanate Health Queen of the Valley Hospital, West Covina, USA; 4 Neurology, University of California Riverside, Riverside, USA

**Keywords:** spinal cord compression weakness, spinal abscess, persistent mssa bacteremia, covid-19 infection, post viral syndrome

## Abstract

Coronavirus disease 2019 (COVID-19), caused by severe acute respiratory syndrome-coronavirus-2 (SARS-CoV-2), has been associated with a plethora of symptoms weeks after the acute infection. While many reports have investigated the novel syndrome of post-acute sequelae of COVID-19, fewer studies have examined post-COVID-19 secondary infections, which may be distinct from typical post-viral bacterial infections due to the multiorgan involvement of COVID-19. This case report aims to highlight a presentation in which a 65-year-old man had COVID-19 and subsequently developed methicillin-sensitive *Staphylococcus aureus *(MSSA) bacteremia with widespread seeding of secondary infections, including abscesses in the hand and paravertebral regions as well as discitis/osteomyelitis of the cervical spine. Further studies are needed to investigate whether an increased susceptibility to unusual secondary bacterial infections is present in post-COVID-19 patients.

## Introduction

As coronavirus disease 2019 (COVID-19) cases have increased since the onset of the global pandemic, a wide variety of complications post COVID-19 has been increasingly recognized. Much research has been published on the post-acute sequelae of COVID-19, a novel phenomenon occurring post-COVID-19 infection in 10-20% of patients [1]. In one systematic review published in 2021, it was found that the five most common symptoms include fatigue, headache, attention disorder, hair loss, dyspnea, among many others, lasting more than two weeks after acute infection [2].

Relatively less attention has been paid, however, to the post-viral bacterial super-infections in COVID-19 cases. Bacterial pneumonias in post-influenza patients have been frequently studied, and are likely facilitated by complex interactions between viruses and the host immune system and disruption of the mucosal barrier within the respiratory tract [3]. As will be discussed in this case report, the multiorgan spread of COVID-19 has potential implications for the widespread seeding of secondary bacterial infections in a post-COVID-19 patient.

## Case presentation

A 65-year-old man presented with complaints of worsening shortness of breath and severe low back, neck, and right hand pain after a mechanical fall three days prior to presentation. The patient had a history of hospitalization for severe COVID-19 pneumonia one month earlier, in January 2022, as well as type 2 diabetes mellitus, chronic obstructive pulmonary disease (COPD) on 2 liters per minute of home oxygen, and obesity.

**Figure 1:**
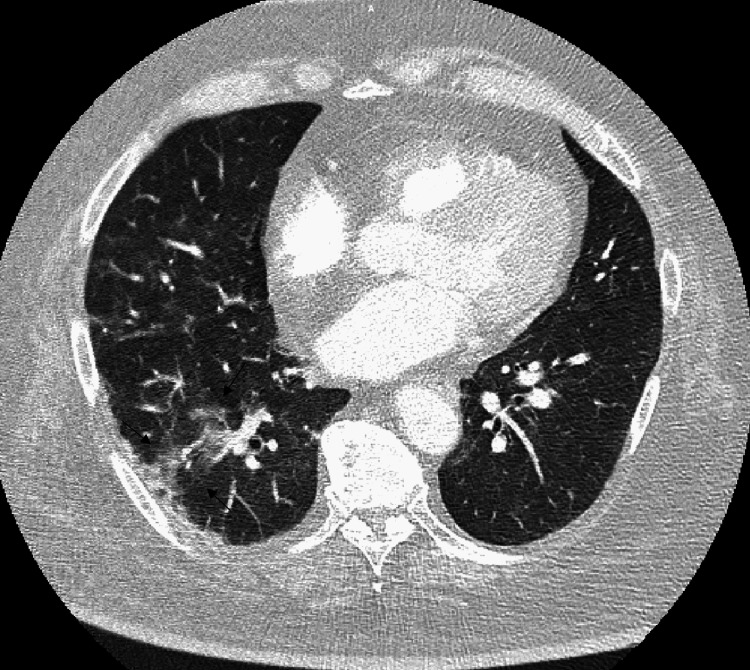
CT imaging of the lungs showing mild ground glass opacities in the right lung.

In the emergency department, the patient was noted to have fever, leukocytosis, and atrial fibrillation with rapid ventricular rate. On physical examination, a 1.5 cm laceration was noted on the right hand with erythema and edema. Neurological exam was grossly negative for acute processes, as the patient demonstrated grossly intact cranial nerves II-XII, intact sensation to light touch throughout his body, 5/5 muscle strength in his upper and lower extremities, no hyperreflexia, and negative Hoffmann’s and Babinski signs. Severe acute respiratory syndrome coronavirus 2 (SARS-COV-2) reverse transcriptase polymerase chain reaction (RT-PCR) swab was negative. Computed tomography (CT) pulmonary angiogram showed no evidence of pulmonary embolism but patchy ground glass densities in the right lung (Figure 1). The patient was started on intravenous (IV) fluids and antibiotics (vancomycin, cefepime, and clindamycin) and an amiodarone drip, and he was admitted to the Definitive Observation Unit (DOU) for severe sepsis and soon transferred to the Intensive Care Unit (ICU) for worsening acute respiratory failure requiring intubation and mechanical ventilation.

The patient’s blood cultures eventually grew methicillin-sensitive *Staphylococcus aureus* (MSSA) in two of two cultures, and his antibiotics were narrowed to nafcillin and clindamycin. CT scans of the patient’s right hand showed abscesses and polymyositis along the palmar thenar bursa, pronator quadratus, and first dorsal interosseous muscle, which received incision and drainage by orthopedic surgery. IV clindamycin was subsequently discontinued.

Due to the patient’s complaints of severe back and neck pain and reported lower extremity weakness, CT scans of the cervical and lumbar spine were performed, which revealed no acute abnormalities. Neurosurgery was consulted and determined that the patient was neurologically stable and had no indication for surgical intervention. Furthermore, the patient was not a surgical candidate at the time due to his sepsis and tenuous cardiopulmonary status. The patient remained persistently bacteremic, and consequently IV antibiotics were changed to daptomycin and oxacillin.

**Figure 2:**
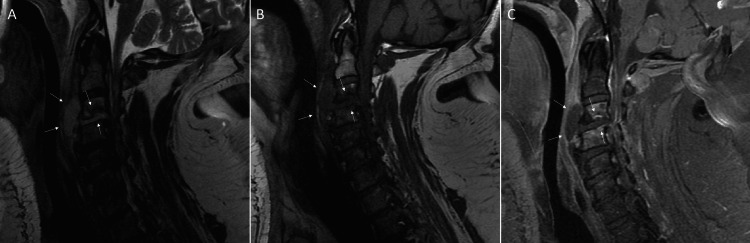
MRI of the cervical spine with (A) fat saturated T2 imaging, (B) T1 imaging, and (C) T1 imaging with gadolinium-based contrast showing evidence of discitis/osteomyelitis centered at C3-4 with adjacent paravertebral abscesses, and suggestion of epidural phlegmon in the anterior epidural soft tissues of C2 and C3.

The patient’s condition gradually stabilized, and he was able to be weaned off vasopressors and sedation. Once more alert, the patient was discovered to have quadriplegia with diminished sensation throughout his body. A STAT (immediate) magnetic resonance imaging (MRI) scan was done on the patient’s cervical spine, which revealed severe C2-7 spinal cord compression, with evidence of multiple sites of infectious processes including discitis/osteomyelitis centered at C3-4, adjacent paravertebral abscesses, evidence of septic arthritis at C3-4, possible osteomyelitis in the vertebral bodies of C5 and C6, and developing epidural phlegmon 2 mm in thickness within the anterior epidural soft tissues of C2-3 (Figure 2). Neurosurgery urgently re-evaluated the patient, who was then taken to the OR for multilevel posterior cervical laminectomies and bilateral foraminotomies. Abscesses were confirmed throughout the epidural space of C2-3 and at the left C3-4 facet joint, which were successfully drained.

The patient’s hospital course was complicated by an episode of bradycardia progressing to pulseless electrical activity (PEA). A Code Blue was called and return of spontaneous circulation (ROSC) was achieved in the patient after administration of epinephrine. A transesophageal echocardiogram (TEE) had been performed earlier, which showed no evidence of intracardiac vegetation. The etiology of the patient’s bradycardia was suspected to have been related to autonomic dysfunction secondary to the patient’s spinal cord compression. As the patient had been on continued antibiotics since admission, he received clearance from Infectious Disease for permanent pacemaker placement.

After pacemaker placement, the patient remained hemodynamically and clinically stable. His surveillance blood cultures subsequently were negative for MSSA bacteremia, and daptomycin was discontinued after two weeks. The patient still required mechanical ventilation and received a tracheostomy placement. After a 36-day hospital course, he was discharged to a long-term acute care facility with an additional six weeks of IV oxacillin. On the day of discharge, the patient was alert and fully oriented, though he still had quadriplegia with very limited sensation inferiorly from his chest.

## Discussion

Viral pneumonias have long been associated with subsequent bacterial super-infections, particularly in the case of influenza pneumonia. Proposed mechanisms of action are multifactorial and include direct epithelial damage by the virus, increased bacterial colonization of both the upper and lower respiratory tracts, and immune dysregulation [4]. These factors, when combined, can create an optimal environment for bacterial super-infections, often caused by *Streptococcus pneumoniae*, *Haemophilus influenzae*, and *Staphylococcus aureus* [5].

While SARS-CoV-2 has predominantly been shown to replicate in the respiratory system, SARS-CoV-2 RNA has also been found to be detected in the kidneys, liver, heart, brain, and blood samples at autopsy, suggesting multiorgan involvement [6,7]. In addition, COVID-19 has been commonly associated with dysfunction of endothelial cells, which play a major role in limiting bacterial dissemination in the systemic inflammatory response caused by bacteremia [8,9]. In this case report, the multiorgan involvement in the patient’s earlier COVID-19 infection, when combined with his other comorbidities, may have contributed to his severe infectious course. The patient appeared to have developed MSSA pneumonia several weeks after his initial COVID-19 infection and subsequently became bacteremic. As a result, his generalized weakness caused him to suffer a mechanical fall, and the following trauma likely further predisposed him towards his discitis/osteomyelitis and epidural abscesses as well as his deep hand abscesses. An alternative interpretation may be that rather than through the respiratory system, the 1.5 cm right-hand laceration provided a direct point of entry for MSSA to enter the systemic circulation, which caused a rapid clinical decline within days. Nevertheless, the severity of the patient's subsequent clinical course is noteworthy and raises the question of whether there exists an increased incidence of severe secondary systemic infections attributable to COVID-19's potential multiorgan involvement. Further cohort studies may better elucidate whether there is indeed a significant difference in relative risk for patients to develop severe secondary bacterial infections following COVID-19 versus other viral respiratory infections such as influenza.

Since the emergence of COVID-19, many reports have been published on the post-acute sequelae of COVID-19, defined as persistent symptoms and/or delayed or long-term consequences of COVID-19 infection [10]. It is estimated that 10-20% of patients testing positive for SARS-CoV-2 remain feeling unwell beyond three weeks, with some feeling symptomatic for months. These complications involve many organ systems and include pulmonary fibrosis, thromboembolic events, myocarditis, nephropathy, and neuropsychiatric sequelae such as chronic malaise, diffuse myalgia, depression, and sleep disturbances [10]. Such neuropsychiatric symptoms may also have contributed to this patient’s generalized weakness and subsequent mechanical fall. Current studies have found promising results in post-infection rehabilitation interventions such as exercise in mitigating the reported neuropsychiatric sequelae of COVID-19 [11].

## Conclusions

It is clear that the management of post-acute COVID-19 deserves additional study. As is commonly seen with influenza-like illnesses and observed in this case report, post-COVID-19 bacterial super-infections remain an important consideration for clinicians, particularly when managing patients with multiple comorbidities. Additional exploration in this topic is warranted due to the novelty of the pathophysiology observed in COVID-19.
